# Parents’ Education Shapes, but Does Not Originate, the Disability Representations of Their Children

**DOI:** 10.1371/journal.pone.0128876

**Published:** 2015-06-08

**Authors:** Fabio Meloni, Stefano Federici, John Lawrence Dennis

**Affiliations:** 1 Department of Philosophy, Social & Human Sciences and Education, Perugia, Italy; 2 Department of Psychology, Catholic University, Milan, Italy; 3 The Umbra Institute, Perugia, Italy; IIT—Italian Institute of Technology, ITALY

## Abstract

The present research tested whether children’s disability representations are influenced by cultural variables (e.g., social activities, parent education, custom complex variables) or by cognitive constraints. Four questionnaires were administered to a sample of 76 primary school aged children and one of their parents (n = 152). Questionnaires included both open-ended and closed-ended questions. The open-ended questions were created to collect uncensored personal explanations of disability, whereas the closed-ended questions were designed to elicit a response of agreement for statements built on the basis of the three most widespread disability models: individual, social, and biopsychosocial. For youngest children (6–8 years old), people with disabilities are thought of as being sick. This early disability representation of children is consistent with the individual model of disability and independent from parents’ disability explanations and representations. As children grow older (9–11 years old), knowledge regarding disability increases and stereotypical beliefs about disability decrease, by tending to espouse their parents representations. The individual model remains in the background for the adults too, emerging when the respondents rely on their most immediately available mental representation of disability such as when they respond to an open-ended question. These findings support that the youngest children are not completely permeable to social representations of disability likely due to cognitive constraints. Nevertheless, as the age grows, children appear educable on perspectives of disability adhering to a model of disability representation integral with social context and parent perspective.

## Introduction

The models of disability are categorical representations from which social relations are developed, built, and understood. They are common structures for making sense of the complex phenomena of disability [1, 2]. As a cognitive organizer, a model of disability helps people to identify and explain social reaction to human, biological, and social diversity. Hence, it offers social frames [3] where expected behaviors and social identities are represented, helping people to make decisions and judgments [4].

To date, the scientific literature has identified three main disability models: individual (taken to include the medical model), social, and biopsychosocial [5, 6]. The individual model is clearly defined in Nagi’s pathological model from the 1960s [7, 8]. However, its relevance was questioned by the social model that was developed within the Marxist dialectical approach of the 1960s and 1970s [5, 9, 10]. Later, those two models were integrated into the biopsychosocial model using influences from transdisciplinary approaches and theories—i.e., integrated complexity [11] and system theory [12, 13] in addition to cybernetics [14]. The biopsychosocial model avoids both the strict biological reductionism of the individual model and the socio-cultural determinism of the social model. The model also uses and finds the most authoritative and practical application in the *International Classification of Functioning*, *Disability and Health* (ICF; [6]).

The scientific community has adopted the individual and social model as alternative and opposite descriptions of human functioning and health conditions [5, 6, 9, 15–17]. Conversely, the biopsychosocial model is often meant as an integration of the two opposing models: individual and social [6]. The individual model claims that disability is a direct consequence of disease, trauma, or other health conditions. Therefore, disability is viewed as “something imposed on top of […] impairments by the way [… disabled people] are unnecessarily isolated and excluded from society” (see p. 4 [18]). Hence, the disability is a problem of the individual, that is, as an individual’s deviation from the biomedical norms of structure or function that requires medical care treatments provided by health professionals. For this reason, the individual model was also defined as a medical model of disability [6, 9, 17, 19]. In disability studies, all models that define disability as being outside of religious, ethical, or aesthetic norms are encompassed within the individual model [20–22]. Therefore, every time we refer to the individual model, we also mean to include the medical one.

Conversely, from the individual model, the social model portrays disability as a political issue, created by the social environment. Physical barriers or social attitudes are considered the origin of disability, preventing access to virtual and real spaces or making social participation difficult for people with diversity in functioning. Consequently, a disability is not an attribute of an individual, but rather a complex collection of conditions, requiring social actions rather than just medical treatments [6].

Finally, since 2001, the ICF has used a biopsychosocial—also called interactive [1]—model of human functioning and health which represents an integration of the two conflicting models (medical and social). To achieve this “synthesis” (see p. 28 [6]), the disability is not a consequence of disease but the outcome of triadic causation of three variables related to human health: health status, environment, and personal factors. In the ICF’s model, these factors are considered dimensions and not determinants of disability, as ‘pathology’ in the individual model [7, 8] or ‘environmental factors’ in the social one [23].

Until the last century, disability research was conducted predominantly—though not exclusively (e.g. rehabilitation theory, epidemiology, and health and social policy)—within a sociological perspective (see the British approach to disability studies: [2, 24–26]) consistent with the dominant paradigm in the social sciences known as the Standard Social Science Model (SSSM; [27, 28]) or social constructionism [29]. The SSSM argues that procedural or algorithmic features, without content, are innate, while mental organization and categorization are heavily influenced by culture [28]. The human mind is little more than a blank slate [28, 30–32] that incorporates a capacity for culture shaped and transformed by social factors [33]. The SSSM influences disability studies such that disability (with its core concepts of diversity and difference) is regarded as a cultural construction process neglecting any element related to the innate cognitive architecture of humans [34].

The present research tested whether beliefs about disability models are transmitted culturally. Whether or not the disability models are exclusively a cultural product, they are only the outcome of a learning process. We surveyed the extent of the cultural transmission of the three disability models (individual, social, and biopsychosocial) by comparing the disability-related descriptions and beliefs of parents and their children. Accordingly, the disability models were considered as dependent variables potentially being affected by the child milieu (family value and education). To the extent that the child-related individual differences in disability models were not totally attributable to the child milieu, we hypothesized that children are not completely permeable to social representations of disability because of cognitive constraints.

A modified version of Evans’s [35] experimental paradigm, originally used to survey the emergence of beliefs about the origin of species in children, was used to test these beliefs. Comparing parent and child beliefs and disability descriptions helped to investigate the extent of cultural transmission of the three disability models (individual, social, and biopsychosocial).

## Background

In the early 2000s, Evans [35–37] investigated the emergence and spread of beliefs regarding the origin of species in both fundamentalist and non-fundamentalist Christian school communities. Using a mixed qualitative–quantitative design, Evans compared the beliefs of children and parents about the origin of species to evaluate if the spread of creationist and evolutionist beliefs in the general population was indicative of cognitive constraints or was simply a function of social forces [35].

Creationism and evolutionism have historically and culturally been viewed as contrasting and polarizing. The same holds true with disability models, which are contrasting perspectives on human beings and the social world. These models have influenced the education of children from the time children enter primary school. While the individual model, much like creationism, seems intuitively plausible [4, 25, 38, 39], the social model, developed as a reversal of the individual model, is as counterintuitive as evolution [25, 40–43].

## Method

### Participants

A total of 76 primary school aged children and their parents (N = 152) participated in this study (S1 File–Dataset). Of this, 46.1% of the children were male (N = 35) and 53.9% were female (N = 41), with a mean age of 8.46 (M = 8.40; F = 8.51; SD (M/F) = 1.46). In addition, 28.9% of the parents were male (N = 22) and 71.1% were female (N = 54), with a mean age of 42.01 (M = 43.23; F = 41.52; SD (M/F) = 5.21). One child and his parent’s data were eliminated because of parent’s illegible writing. All of the children in the sample attended state school. The Institutional Review Board of the Department of Philosophy, Social & Human Sciences and Education at the University of Perugia reviewed and approved this study. The parents provided their written informed consent to participate in this study for themselves and on behalf of the children enrolled in our study. The study presented “no more than minimal risk.” In order to replicate Evans’ [35] procedure, the children were divided into two age groups: 6–8 years old (N = 38) and 9–11 years (N = 38).

### Materials and procedure

Four questionnaires were administered to both the children and the parents. The questionnaires maintained the same format as the questionnaires that Evans [35] used, i.e., item number, item type (open or closed questions), Likert scale ranges, and open-ended questionnaire coding system. However, the item content, instructions, and questionnaire stimuli were modified (see S2 File–Details of Measure for a detailed description of the measures, coding, and procedures). The open questions were created to collect uncensored personal explanations of disability and diversity, whereas the closed questions were designed to elicit a response of agreement, presented in a forced–choice model, using explanations built on the basis of the three disability models. The latter techniques act as scaffolds [44], reminding respondents of the explanations according to the publicly present disability models, of which they may not have access to without such support.

The children were interviewed at their home separately from their parents. Comprehension of the concept of “disabled vs. normal person” was verified via a semantic discrimination task. The children’s explanation of disability (*Disability Explanation*) was assessed using one open-ended and one closed-ended questionnaire, while activities or interests (*Child’s Interests and Activities*), as well as agreement with health and disability statements (*Child’s Disability Knowledge*), were assessed using two closed-ended questionnaires (S2 File Details of Measures). Statements on health and disability were constructed on the basis of false beliefs (stereotypes) and true content (knowledge).

After checking the child’s questionnaires, one child’s parent completed three closed-ended and one open-ended self-administered questionnaire. The open-ended questionnaire and one of the closed-ended questionnaires were based how they as parents explained the concept of disability to their child (*Disability explanation*). Child activities or interests that the parent encouraged (*Parent’s interests and activities encouraged*) and how they explained diversity to their child (*Parent’s education about diversity*) were assessed using two closed-ended questionnaires (S2 File–Details of Measure).

## Results

### Disability Explanation—Open-Ended Questionnaire

**Fig 1:**
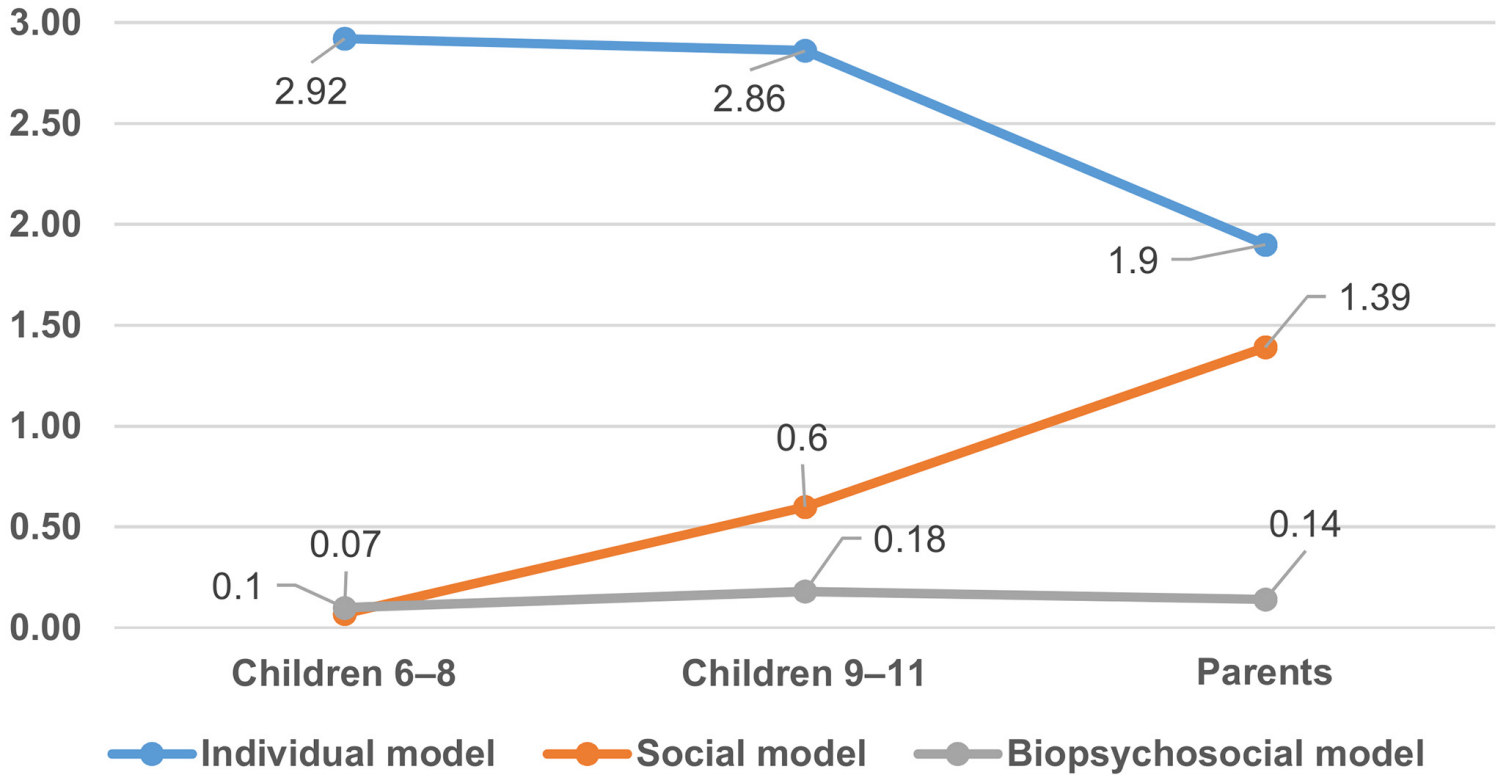
Disability Explanation—Open-Ended Questionnaire. Mean frequency preference for the three disability models by age group.

For the present article, in order to force data in the room of a scientific article, we decided to report the only quantitative data, reserving a qualitative analysis for a future article. The disability model frequency scores were analyzed in a 3 (disability model: individual, social, biopsychosocial) x 3 (age group: children 6–8 years, children 9–11 years, parents) mixed-design analysis of variance (ANOVA). Results are shown in Fig 1. The analysis revealed a main effect for disability model, F(2, 148) = 402.257, p < 0.01, and a significant interaction, disability model x age group, F(4, 296) = 15.678, p < 0.01. Three univariate ANOVAs clarified the nature of the interaction by demonstrating a significant effect of age for the individual, F(2, 149) = 27.212, p < 0.01, and the social model, F(2, 149) = 27.146, p < 0.01. Fisher’s post-hoc LSD showed that children were more likely to use the individual model (p < 0.01) while parents were more likely to use the social model (p < 0.01).

### Disability Explanation—Closed-Ended Questionnaire

**Fig 2:**
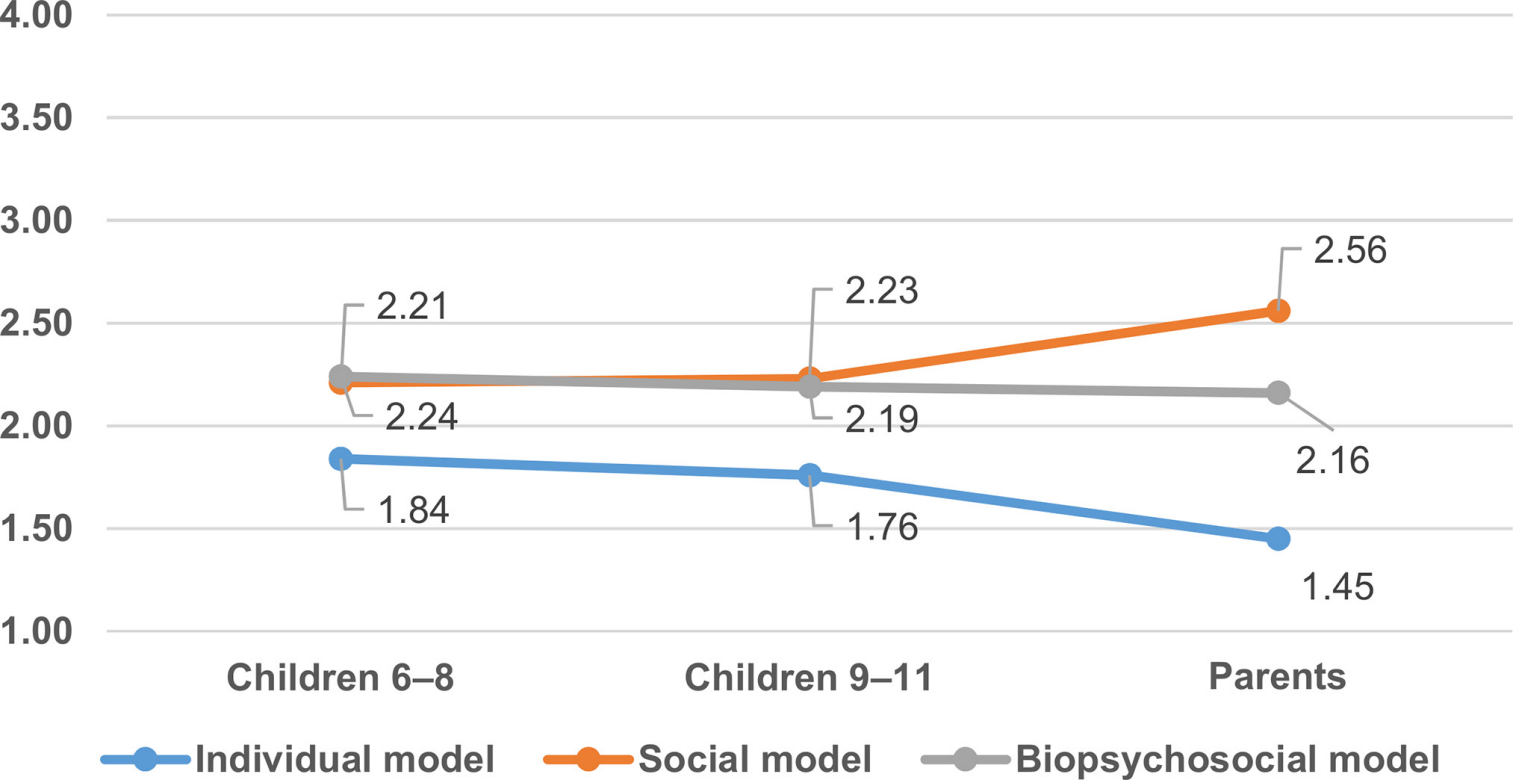
Disability Explanation—Closed-Ended Questionnaire. Mean frequency agreement for the three disability models by age.

The disability model agreement scores were analyzed in a 3 (disability model: individual, social, biopsychosocial) x 3 (age group: children 6–8 years, children 9–11 years, parents) mixed-design analysis of variance (ANOVA). Results are reported in Fig 2. A main effect for disability models emerged F(2, 147) = 78.828, p < 0.01, and a significant interaction: model of disability x age groups, F(4, 294) = 12.238, p < 0.01. Three univariate ANOVAs (one way) were conducted on agreement scores. A significant effect of age for the individual model, F(2, 148) = 13.513, p < 0.01, and the social model, F(2, 149) = 6.912, p < 0.01, emerged, while none was found for the biopsychosocial model. Fisher’s post hoc LSD showed that children’s agreement for the individual model was significantly higher than their parents (p < 0.01). Parents’ agreement for the social model was significantly higher than their children (p < 0.01).

Parents preferred the social model and significantly more so compared to children aged 6–8 years (p < 0.01) and 9–11 years (p < 0.01) whereas no significant difference between the two children’s groups was found. No significant age difference emerged regarding the biopsychosocial model.

### Parents’ Education about Diversity: Was the disability a special case?

The parental endorsement scores for the disability models were analyzed in a 3 (disability model: individual, social, biopsychosocial) x 2 (group of parents: parents’ children of 6–8 years and 9–11 years) x 2 (diversity condition: disabled and non-disabled) mixed-design analysis of variance (ANOVA) with disability model as the repeated measure. A main effect for model of disability F(5, 70) = 28.853, p < 0.01, and no significant interaction for children age were found. Planned comparisons revealed that the biopsychosocial model was preferred to the social, t(75) = -7.139, p < 0.01, and individual model t(75) = -11, p < 0.01. Parents of children from both age groups significantly preferred the social model to explain stimuli showing non-disability/diversity conditions compared to the disability/diversity conditions, t(37) = -2.149, p < 0.05.

### The custom complex: knowledge and stereotypes on disability, interest, and activities of the children

In order to analyze the complex world of culture and habits of children, one-way ANOVAs were conducted on the two age groups for each of the following three scales: (i) the Child’s Disability Knowledge scale, (ii) the Child’s Interests and Activities scale, and (iii) the Parent’s Interests and Activities Encouraged scale. Children 9–11 years have significantly more knowledge, F(1, 74) = 9.218, p < 0.01, and fewer stereotypes, F(1, 74) = 6.948, p = 0.01, about disability than children 6–8 years. Parent’s encouragement of interest or cultural activities was significantly higher in older children, F(1, 74) = 5.365, p < 0.05. No significant interaction was found between parent’s encouragement of interest or cultural activities and child’s disability knowledge.

### The typology of the beliefs: the explanatory coherence

**Fig 3:**
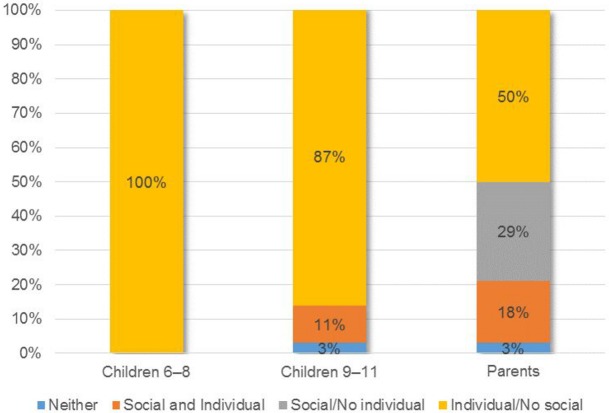
Frequencies of the composite models created on the open-ended responses.

**Fig 4:**
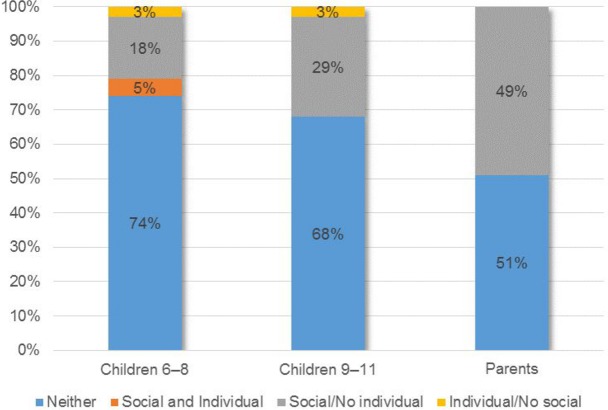
Frequencies of the composite models created on the closed-ended responses.

Reasons why people with and without disabilities encounter difficulties in life differed significantly across age groups (children 6–8 years, 9–11 years, and parents). Results are at the group level and not at the individual difference level. In order to draw a more accurate difference profile, two composite measures of consistency between the models were built, by combining the responses related to the individual and social model in the open-ended and closed-ended questions (S2 File–Details of Measure). The composite measure created on the frequency scale of the open-ended responses range from a minimum to a maximum value of -3 to +3, where the highest score indicated a higher frequency of use of the individual model. Based on these consistency measures, typologies were then created in which participants were divided into four groups: (i) social/no individual model; (ii) individual and social model; (iii) individual/no social model; (iv) neither (Figs 3 and 4).

Children aged 6–8 years (N = 38) preferred exclusively the individual model and all children significantly more than parents (p < 0.01). No children adhered to the social/no individual model, conversely to the parents who strongly (p < 0.01) preferred it. The social and individual model was significantly (p < 0.05) preferred by parents compared to their children. The class “neither” gathered very few preferences among groups (Fig 3). This last result was challenged by the adhesions calculated on the basis of answers to the closed-ended questionnaires, where the “neither” class received the highest consent between groups without difference among groups (Fig 4).

In addition, the adherence to the individual model collapsed vertically with respect to the open-ended in all groups, with no differences among them, whereas the number of those who preferred the social model increased with age (p < 0.05).

Pearson’s correlation between the two composite measures (4 composite models: neither, social and individual, social/no individual, individual/no social x open- and closed-ended questionnaires) revealed: (i) a significant (p = 0.05) positive correlation between individual/no social in open-ended and “neither” in closed-ended and (ii) negative correlation (p < 0.05) between individual/no social in open-ended and social/no individual in closed-ended.

### Children’s and parents’ models and custom complex variables

In order to test the relationship between the children’s custom complex—i.e., the interconnected practices, beliefs, and values in which the child is embedded [45]—and their disability model choices, correlations between children and parents’ model choices and (i) Child’s Disability Knowledge, (ii) Child’s Interests and Activities scale, and (iii) the Parent’s Interests and Activities Encouraged scales were carried out. Two consistency scales for parental models for each type of answer (open and closed) were created ranging from -3 to +3. Scores were obtained by subtracting the values obtained on the social model by the ones of the individual model. We proposed, in the Introduction, that the two models are theoretically polarized in a unique conceptual line so that the more an individual prefers a social model of disability the less likely they are to prefer the individual, and vice-versa. Two separate correlations were carried out for each of the two children age groups (6–8 years and 9–11 years) with the new two consistency scales and the custom complex variables.

As shown in Table 1, children 6–8 years’ preference (open-ended) for the individual model negatively correlated with parental encouragement of religious (p < 0.05) and social activities (p < 0.05). Children 6–8 years’ agreement (closed-ended) for the individual model positively correlated with their appreciation of social activities and interests (p < 0.05). Children 6–8 years’ agreement for the social model correlated positively with their knowledge level about disability (p < 0.01) and with their interests and preference for activities of general type (p < 0.05). The more the parents agreed the individual model, the more the children of 6–8 years were interested in religious activities (p < 0.01) and the less in activities related to body and health (p < 0.05).

**Table 1:** Correlation between children 6–8 years’ custom complex and their disability model choices.

Interests and Activities	Children 6–8 years’ choices for individual and social model			
	Individual model in open-ended	Social model in open-ended	Individual model in closed-ended	Social model in closed-ended
Child’s Disability Knowledge: Knowledge items				.541**
Child’s Interests and Activities: Social activities (helping a classmate with homework, helping to push a wheelchair, etc.).			.394*	
Child’s Interests and Activities: General activities (e.g. reading stories, visiting a museum, etc.)				.344*
Parent’s Interests and Activities Encouraged: Religious activities (going to church or other worship, praying or meditation places, etc.)	-.358*			
Parent’s Interests and Activities Encouraged: Social activities (participation in voluntary groups, attending study groups or extra-curricular activities, etc.)	-.326*			

To simplify the readability of the table, only those variables and scores whose Pearson’s correlation value was significant are reported.

**. Correlation is significant at the 0.01 level (2-tailed).

*. Correlation is significant at the 0.05 level (2-tailed).

As shown in Table 2, children 9–11 years’ preference for the social model inversely correlated with their parent’s agreement (closed-ended) for the individual model (p < 0.05), while parental’ preference (open-ended) for the individual model positively correlated with children 9–11 years’ agreement for the individual model (p < 0.05). The agreement (closed-ended) for the individual model in children 9–11 years also correlated positively with their stereotype level about disability (p < 0.05) and their interest in social activities (p < 0.05) and negatively with general interests and activities (p < 0.01). Agreement (closed-ended) for the social model positively correlated with a greater knowledge of disability (p < 0.05) and with interests and activities of a religious nature (p < 0.05).

**Table 2:** Correlation between children 9–11 years’ custom complex and their disability model choices.

Interests and Activities	Children 9–11 years’ choices for individual and social model		
	Social model in open-ended	Individual model in closed-ended	Social model in closed-ended
Child’s Disability Knowledge: Knowledge items			.378*
Child’s Disability Knowledge: Stereotype items		.383*	
Child’s Interests and Activities: religious (e.g. reading stories from the Bible, saying prayers, etc.)			.325*
Child’s Interests and Activities: Social activities (helping a classmate with homework, helping to push a wheelchair, etc.)		.340*	
Child’s Interests and Activities: General activities (e.g. reading stories, visiting a museum, etc.)		-.569**	
Parental’ preference for the individual model in open-ended		.331*	
Parent’s agreement for individual model in closed-ended	-.338*		

To simplify the readability of the table, only those variables and scores whose Pearson’s correlation value was significant are reported.

**. Correlation is significant at the 0.01 level (2-tailed).

*. Correlation is significant at the 0.05 level (2-tailed).

## General Discussion

### Disability Explanation—Open-Ended Questionnaire

Considering the open-ended questionnaire, the individual model is most commonly used by all age groups but decreases with age while preferences for the social model increase with age. These results are in line with a previous study where the individual model was preferred to the social model via the Implicit Association Test [4]. In contrast to the individual model, the use of the social model significantly increases from the youngest to the older. In other words, the younger the respondent is, the greater the preference for the individual model. This prompts us to believe that the strong polarization between individual and social model [5, 15–17] is a cultural construct rather than a cognitive constraint: One births “individual” and dies “social.” The ontogenetic evolution of the social model seems to mirror the historical development of the disability models; whereas the individual model crosses throughout the human history with its several faces (religious, ethic, aesthetic, medical, biological) [20–22, 46], the social model developed recently in Britain in opposition to the individual one [18, 25]. By adopting a social perspective on disability (social model) as they age, people provide a more complex explanation of disability, although not at the complexity level of the biopsychosocial model [6], which is not preferred by any age group.

### Disability Explanation—Closed-Ended Questionnaire

Considering the closed-ended questionnaire, preference for the social model increases with age whereas preference for the individual model decreases with age (Fig 2). The most striking difference when comparing the open- and closed-ended questionnaires is that the mean agreement for the social (and biopsychosocial) model is significantly higher than for the individual model for all age groups. What happened in the shift from the open- to closed-ended questions, administered to the same respondents in a span of a few minutes from each other? We think that people used two cognitive processes elicited by different contexts hinged on two distinct testing procedures. In the open-ended questions, the task was to provide a personal opinion on why a man in a wheelchair (or a blind woman, or an autistic child, or an able-bodied woman) has difficulties in life. In this case, respondents recall from memory and process a veridical explanation, as consistent as possible, by relying on their most immediately available mental lexicon. In this way, the respondent, not trained in disability study and models, is more subjected to an automatic cognitive process [47, 48]. As found in our previous experiment [4], on an implicit level, people strongly and stereotypically associate disability with concepts that characterize the individual model rather than the social one. In contrast, in answering the closed-ended questions, the respondent has the opportunity to evaluate statements built to represent all the main theoretical features of the disability models. Respondents were asked to rate statements often mutually contradictory and sometimes politically incorrect and not socially acceptable. In this case, they tended to provide more socially desirable answers [49].

The most socially acceptable statements in Italian culture are, without doubt, wording that reflects the social model of disability rather the individual model [4, 39]. The Italian legislature has promoted social inclusion of people with disability (Law 517/77) for more than 40 years; meanwhile, twenty-two years of integration of students with disabilities at all school levels (Law 104/92) [50] have widespread a social model of disability. Maybe for these reasons, even the children found the statements reflecting the social model (e.g., “[Giovanni has difficult in life because] he encounters lots of obstacles”) more agreeable than those related to the individual model (e.g., “[Giovanni has difficult in life because] he’s ugly”) (S2 File Details of Measures). This suggests that even if the attractiveness of the individual model remains persistent, it is possible that other disability representations accompany and overlap it. Briefly, the representation of disability is, to some extent, improved upon education.

Notwithstanding the striking emergence of the social model in the closed-ended questionnaires, though, the endorsement of all age groups did not reach a properly positive level. In fact, the highest score was for one of the parents on the social model (M = 2.56), which was little more than the mid-point of the scale. The scores of the children are all below the mid-point value. Hence, even though it is more politically correct and socially desirable, the social model is not entirely convincing. The (Italian) cultural context does not seem, therefore, to be sufficiently strong to neutralize individual cognitive constraints. In addition, the results of the open- and closed-ended questionnaires suggest that the theory on disability can be learned: the growing children endorse parents’ explanations regarding diversity.

### Parent’s Education about Diversity

Interestingly, the child’s age did not influence parental explanations of disability. Parents preferred to explain diversity and disability to their children by choosing from among expressions reflecting the biopsychosocial model (e.g., “We are all a bit different; differences much depend on where we are if we function as well or not”) (S2 File–Details of Measure). For parents, the disability (a person with a physical disability and a person with Down’s syndrome) did not appear to be a “special condition” when it was compared to other kinds of diversity (a boatload of migrants, two gay men kissing, and a pelican covered in oil) (S2 File Details of Measures). The only significant difference concerned the use of social model that appears to be preferred by parents when explaining situations of diversity with respect to human health conditions.

Furthermore, as we found in the closed-ended questionnaires, when the respondent was encouraged to choose from among different statements built on the three models (individual, social, biopsychosocial), the individual model lost salience compared to the social and biopsychosocial models. The individual model, as a cognitive organizer at an implicit level [4, 51], was overwhelmed by cultural constructs provided by the social-cultural context when the respondent accessed an explicit and reflexive thought.

#### The custom complex

The complex of cultural elements, habits, interests, and activities of the children showed no significant differences between the two age groups of subjects. The cultural environment constructed by parents, mainly through the encouragement of certain interests or activities, was not different for the two groups, except for a greater pressure to engage in cultural activities carried out by parents of the older children. Instead, significant differences emerged on the levels of knowledge and stereotypes held by children. In both age groups, the level of knowledge about disability was higher than the levels of stereotypes. In addition, knowledge increased and stereotypes decreased with the age growing. This result is coherent with the studies that claim the origin of stereotypes toward people with disability is due to a cognitive immaturity [52, 53].

### The typology of the beliefs

The analyses performed on the open- and closed-ended questionnaires showed the prevalence of one model (open-ended: individual model; closed-ended: social model) within and between groups. As group results obscure individual differences [35], it is not clear whether, for parents and children, half preferred individual model and half social model or whether most endorsed a mixed model (individual and social). For this reason, the composite measures allow one to investigate individual differences within groups in more detail. The composite measures built on the open-ended questionnaires showed that the vast majority of respondents firmly chose a singular model and not a mixed one. Almost all of the children preferred the individual model, whereas 50 percent of the parents preferred the individual model, 30 percent preferred the social model, and only 20 percent preferred the mixed model (individual and social). Results of the composite measures on the closed-ended questionnaires demonstrated that the mixed model was not preferred. In contrast to the open-ended questionnaire, a high percentage of respondents (≈ 70% children; ≈ 50% parents) did not agree with either of the two models (neither), whereas the remaining part (≈ 20% 6–8 years; ≈ 30% 9–11 years; ≈ 50% parents) agreed with the social model. The lack of preference for a mixed model could be due to the inherent cognitive dissonance between the individual and the social model. What has been affirmed theoretically and politically [5, 6, 15–17], namely, the dichotomous nature of those two models has been confirmed in this research at a psychological level.

The correlation between the two composite measures again revealed an effect of social desirability: The sharp adhesion to the individual model in the open-ended questionnaires seems masked behind the “neither” agreement in the closed-ended questionnaires.

### Children’s and parents’ models and custom complex variables

Whereas there was no correlations between the children of 6–8 years and their parents about the disability representations, correlations emerged between the children of 9–11 years and their parents. Children, as they grow older, tended to align with the viewpoints of their parents. The parental education permeated cognitive constraints, dominated by the individual model, by injecting more cultural representation of disability, i.e., the social model. Significant correlations were evident between the children’s models of any group and their levels of knowledge and stereotypes: A high level of knowledge was positively correlated with the expression of the social model, whereas a high level of stereotypes correlated with a greater appreciation of the individual model. These findings confirm, at a psychological level, what was extensively investigated from a sociological perspective by disability studies since the late 1960s (for an authoritative and up-to-date overview of the main issues in the field of disability studies around the world today Watson and colleagues [26]), namely, the effect of custom complex variables on child’s disability attitudes and stereotypes (see, among many others [54–57]).

## Conclusions

The present research verified whether children’s disability representations are influenced by cultural variables (e.g., social activities, parent education, and custom complex variables) or by cognitive constraints. At an early age, children have a disability representation characterized by a vision of the person with disability mainly as sick and whose diversity is strictly connected to health. This early disability representation of children is consistent with the individual model of disability and independent from parents’ disability explanations and representations. This finding supports that children are not completely permeable to social representations of disability because of cognitive constraints. Nevertheless, children appear educable on perspectives of disability adhering to a model of disability representation integral with social context and parent perspective. This is evident because the children tend to espouse their parents representations as they grow up.

Another cultural effect on children is observable from the stereotype decreasing in correspondence with an increasing level of knowledge about disability. Children’s cognitive maturity reduces stereotypes toward people with disability. These findings are also in line with those of the study previously carried out by the authors with the Implicit Association Test [4]. In this study the authors found that, on an implicit level, disability is strongly associated with a negative and unpleasant dimension of existence (i.e., bad and ill) with concepts that characterize the individual model. In fact, the individual model was found, in the present research, universal in children and maybe more developmentally primitive—i.e., it is acquired very early or is innate and provides for the development of all other concepts [58]. For all the children, people with disability are disabled because they are sick. Moreover, the individual model remains in the background for the adults too, emerging when the respondents rely on their most immediately available mental representation of disability or to an automatic cognitive process, such as when they respond to an open-ended question. In her survey of 311 parental narratives about disabled children, Avery [59] found that “the vocabulary spoken in the family home might serve to reinforce the inscription of the disabled child as a ‘patient’, and might promote a ‘custodial’ view of disability.” Although from a perspective of social constructionism—i.e., by bringing back to social determinants the disability explanations adopted in the parental narratives—however, Avery found the same pervasiveness of the individual model in the adult speeches.

Future research might overcome some limitations of the present study. These include, for example, increasing the sample size by also recruiting preteens (12 to 14 years olds) to better investigate the evolution of the correlation between parents’ and children’s models. This widening of the sample would permit observation about whether children’s disability models overlap more precisely with those of their parents as they grow up to confirm the trend of a certain perviousness of the disability representations to education. Finally, the present research reflects the Italian socio-cultural context, where the pressure to include disabled people in social life is a political and legal norm but not yet widespread in the social fabric, indeed. It would be interesting to see how in other socio-cultural contexts the disability models feeds people’s narratives on disability.

## Supporting Information

S1 File Dataset. Dataset of 152 children (n = 76) and their parents (n = 76) used in this study.
(XLS)

S2 File Details of Measure. Detailed description of the measures, coding, and procedures.
(DOC)
