# Supplementary material for: Parents’ Education Shapes, but Does Not Originate, the Disability Representations of Their Children
Source: PLoS One. 2015 Jun 8;10(6):e0128876. doi: 10.1371/journal.pone.0128876 (PMC4459974; doi:10.1371/journal.pone.0128876)
Supplement: S2 File — Detailed description of the measures, coding, and procedures. (DOC) [file pone.0128876.s002.doc]

**Details of Measures, Procedures, and Coding**

**(Supporting Information S2)**

**Measures’ Description**

**Semantic Discrimination Task**

The Semantic Discrimination Task consists of presenting the child with 6 stimuli (2 photos of people with disabilities, 2 photos of people without disabilities, and 2 words, “handicapped” and “normal[[1]](#footnote-2)”) that must be correctly placed in two baskets. One basket was labeled with a printed tag, “disabled,” and the other with the printed tag, “normal,” and placed in front of the child. The child passed the test when he or she could understand the difference between the semantic categories identified by the stimuli. The test was repeated until the child had either demonstrated that he or she correctly discriminated between the stimuli or was clearly unable to do so. The purpose of the test was to ascertain the child’s understanding of the term, *disabled*.

**Parent’s Education about Diversity**

This questionnaire consists of 12 randomly repeated statements (4 related to the individual, 4 to the social, and the other 4 to the biopsychosocial model) presented with each of five image stimuli. Those images depicted individuals with clearly visible disabilities or belonging to social categories that referred to diversity: (i) a person with a physical disability, (ii) a person with Down’s syndrome, (iii) a boatload of migrants, (iv) two gay men kissing, (v) a pelican covered in oil (Figure A). For each of those 12 statements, participants were asked to indicate how likely they were to use that image in an explanation to their child’s question, “What is disability?” The answers were given on a five-point scale (1 = never; 5 = very likely).


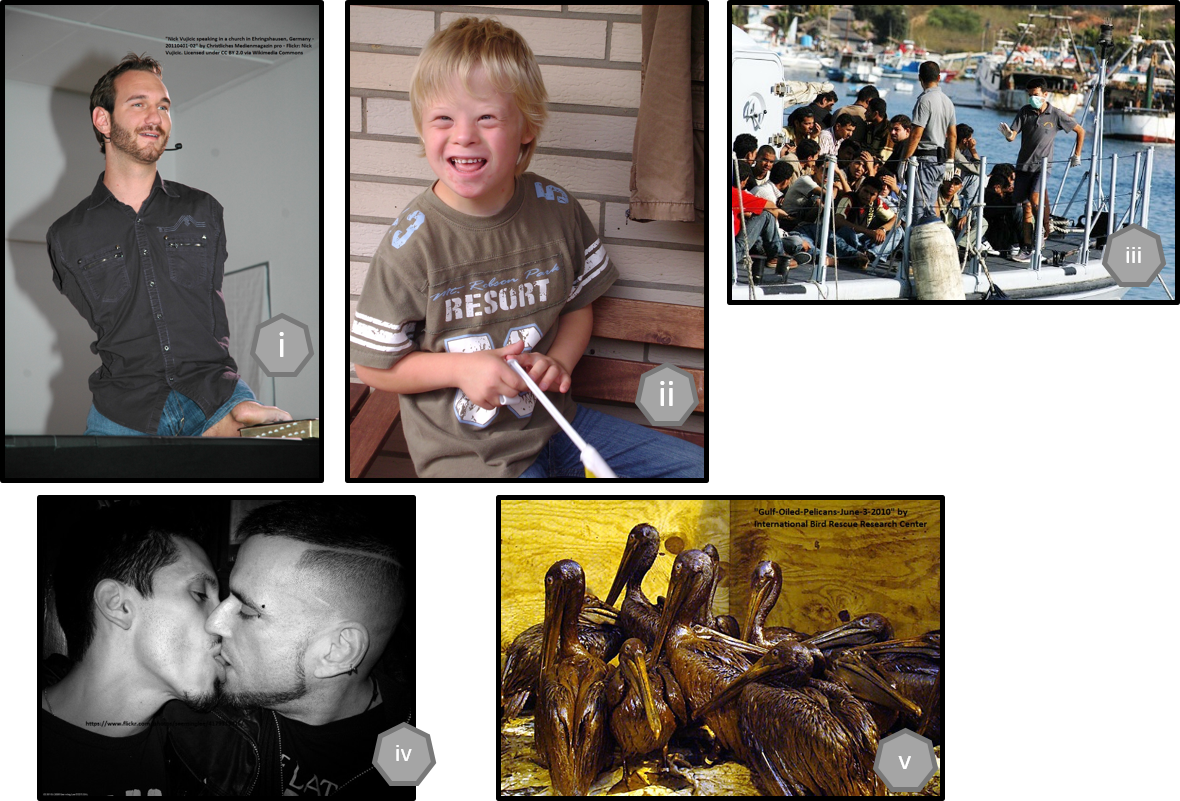


**Fig. A.** **Images of Parent’s Education about Diversity**. From left to right: (i) a person with a physical disability, (ii) a person with Down’s syndrome, (iii) a boatload of migrants, (iv) two gay men kissing, (v) a pelican covered in oil

**Disability Explanation—Open-ended Questionnaire**

Participants were presented one at a time with four images depicting a man in a wheelchair, a blind woman, an autistic child, and an able-bodied woman (Figure B).


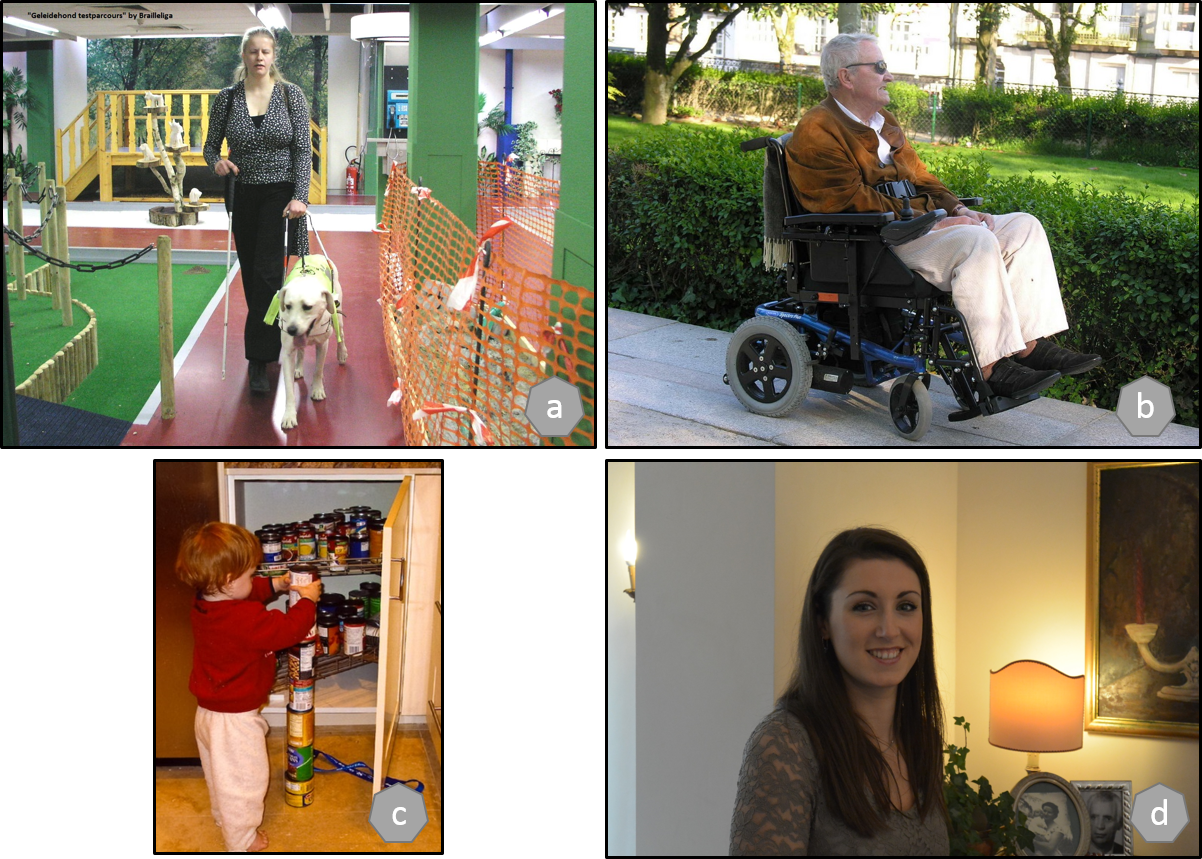


**Fig. B.** **Images of Disability Explanation—Open-Ended Questionnaire**. From left to right: a) man in a wheelchair, b) blind woman, c) autistic child, and d) able-bodied woman.

For each image, participants had to answer the question: “Why does this person have difficulties in life?” Children answered orally and their answers were recorded, whereas parents wrote them in the appropriate spaces on the questionnaire. Each response was then classified by assigning it to one of nine categories of disability models: (i) ethical model, (ii) aesthetic model, (iii) religious model, (iv) medical model, (v) environmental model, (vi) socio-relational model, (vii) biopsychosocial model, (viii) other, (ix) I don’t know. These nine categories were based on the following criteria:

1. *Ethical Model*: We classified any expression in which the individual was considered morally or ethically responsible for his or her disability condition. The ethical model is different from the religious model (see below) because the judgment about an individual’s goodness/badness, responsibility/irresponsibility is not heteronomous, i.e., entrusted to life, God, or forces other than the human being. An expression such as “because he is gay” can be classified in the social and socio-relational model if the expression has been used within a context of social prejudice; it fits within the ethical category if it expresses a judgment of individual conduct.
2. *Aesthetic Model*: Any expression that judged an individual from his or her appearance, i.e., beauty/ugliness, was classified into this model. Not falling into this category were judgments related to social class or economic conditions (poverty, marginalization, employment status, social or political class), which instead fell within the social model.
3. *Religious Model*: We classified any expression which referred the responsibility for the disability to an external spiritual, vital, or religious force: God, Life, Karma, Nature, Fate, and every other human characteristic “according to nature.” This category includes all sorts of popular maxims, aphorisms, sayings, and proverbs, which refer to heteronomous forces and not to the individual’s or society’s responsibility, as well as beliefs about the naturalness of race, gender, sexual orientation, and skin color. Not falling into this category are all judgments that refer the condition of disability to human or social responsibility.
4. *Medical Model*: We classified any expression that referred the causes of disability to an individual health state. This category also included judgments about any individual dysfunction. Clear references to ethical judgments in which the responsibility stems from ethical conduct and not on the health condition did not fall into this category.
5. *Environmental Model*: All expressions that attributed the condition of disability to factors beyond the individual, such as architectural and cultural environments (barriers, rules, regulations, etc.), were classified into this model. The passive construction of a phrase or a description of a condition suffered by individuals with a disability was usually a good indication of judgments belonging to this model. External causes classifiable as religious or related to social attitudes and cultural prejudices that characterize human relationships did not fall into this category.
6. *Socio-Relational Model*: This model shared all the features of the previous one, but it made explicit reference to the attitudes and prejudices that characterize human social relationships. External causes that did not fall into the socio-relational model were classifiable as barriers, rules, and regulations.
7. *Biopsychosocial Model*: This was the least common class and difficult to detect. Since it referred to a composite model, it involved an articulation of a complex interaction of the medical, environmental, and socio-relational models with a clear reference to the individual functioning (health or disease). It is not to be confused with the simple simultaneous coexistence of medical, environmental, and socio-relational models, as they are not put in a clear multifactorial interaction, remaining as juxtaposed and independent. Clear exclusion criterion was the presence of any reference to ethical, aesthetic, or religious elements, so any holistic approach that made clear reference to maxims, proverbs, aphorisms, sayings, or beliefs should have been classified in an ethical model and not in the biopsychosocial one.
8. *Other*: Any complete expression not attributable to one of the previous models.
9. *I don’t know*: This must be clearly expressed as ignorance. It was usually more common in children than in adults. It is not to be confused with the denial of disability that fell within the category “Other.”

According to our classification, the first four categories on the previous list represented different declinations of the individual model, whereas five and six were related to the social model (see the Introduction in the main text).

**Disability Explanation—Closed-Ended Questionnaire**

This questionnaire (one for parents and one for children) investigated explanations about disability that the participants gave when forced by preset choices (closed-ended answers). The stimuli used were the same as for the open-ended questionnaire. For each, the participants indicated on a seven-point scale their agreement or disagreement with eleven statements randomized for each of the four stimuli. All statements provided an explanation for the fact that the person, depicted or described, meets difficulties in life. The stimuli were presented to parents in a fixed order, whereas the children randomly select one at a time from a bag. Children indicated how much they agreed or disagreed with each statement on a card with a four-point scale. On the card there were four expressive faces indicating either disagreement (1, a lot; 2, a little) or agreement (3, a little; 4, a lot). Children should first have been trained to use the card. If any question elicited an “I don’t know” response, the question was repeated one more time.

**Parent’s Interests and Activities Encouraged**

Parents evaluated the importance of 25 potential children’s activities or interests by means of a five-point scale. Activities were grouped into five sets: music (going to a concert, listening to music, etc.), religious (going to church or other worship, praying or meditation places, etc.), sports (going to a sporting event, physical activities at school, etc.), culture (visiting a museum or going to conferences, reading books, etc.), social (participation in voluntary groups, attending study groups or extra-curricular activities, etc.). Activities were presented in a fixed order; the only constraint was that two activities belonging to the same conceptual set did not appear consecutively.

**Child’s Interests and Activities**

Children participated in four tasks in which they were randomly shown five pictures of specific interests and activities common to children in this age range (6–11 years). They ranked the pictures in order from those they liked the most to those they liked the least. Each interest was then assigned a number from 1 (the least) to 5 (the most) for each task. A five-point scale (1, never; 3, once or more a year; 5, once or more a week) was used to indicate how often they engaged in each of the five activities. Conceptually related items from these tasks were grouped into 3–5 items per group and into four measures of interest (range, 1–5): (1) religious (e.g., reading stories from the Bible, saying prayers, etc.); (2) human body (e.g., playing Operation™ [Hasbro, Inc. Pawtucket, RI, U.S.A.], playing doctor, etc.); (3) social activities (e.g., helping a classmate with homework, helping to push a wheelchair, etc.); (4) general activities (e.g., reading stories, visiting a museum, etc.).

**Child’s Disability Knowledge**

Children were given 16 randomly ordered statements on health and disability: 8 were true (knowledge) and 8 were false (stereotype). Children were shown the previously described agree-disagree card with the same four-point scale and it was possible to designate intermediate points to indicate how much they disagreed or agreed with each statement.

**Coding**

**Disability Explanation—Open-Ended Questionnaire**

Coding of the open-ended questions was done by two trained coders on disability models but who were blind to both the nature of the hypotheses and the participants’ demographic information (gender, age, education, etc.). All disagreements were subsequently resolved in discussion. The scoring procedure followed the criteria indicated in the illustrated Table A. When the presence of at least one of the ethical, aesthetic, religious, or medical models was detected, then the presence of the individual model had to be checked. When the presence of at least one of the socio-environmental or socio-relational models was detected, then the presence of the social model had to be checked. The light-gray bars, therefore, simply report the presence or absence (1-0) of the model for each condition of disability, regardless of the number of occurrences of each subcategory for each condition (Table A).

**Table A: Scoring example of the Disability Explanation – Open-Ended Questionnaires.**

| TYPE OF DISABILITY | INDIVIDUAL MODELS | | | | Individual model scores | SOCIAL MODELS | | Social model scores | Biopsychosocial model  scores |
| --- | --- | --- | --- | --- | --- | --- | --- | --- | --- |
|  | Ethical | Aesthetic | Religious | Medical |  | Environmental | Socio-relational |  |  |
| Motor | X |  |  |  | X |  |  |  |  |
| Sensory |  |  |  |  |  | X |  | X |  |
| Intellectual |  |  | X | X | X |  |  |  |  |
| Totals | | | | | 2 |  |  | 1 | 0 |

The scores for individual, social, and biopsychosocial models simply report the presence (X) of the model for each condition of disability, regardless of the number of occurrences of each subcategory for each condition. The total obtained for each macro model (gray row) ranges from 0 to 3, where 0 indicates the absence of any explanation attributable to a disability model (individual, social, and biopsychosocial) and 3 indicates that the model was used in all type of disability (moto, sensory, intellectual): 0 indicates the lack and 3 the maximum strength of an explanatory model of disability.

**Disability Explanation—Closed-Ended Questionnaire**

Each statement might obtain a score ranging from a minimum of 1 to a maximum of 4. The score obtained by each model―individual, social, and biopsychosocial―has been determined from the average score of each statement multiplied by the number of statements about the model.

**The Typology of the Beliefs: The Explanatory Coherence**

To draw up a more accurate profile that is faithful to the individual differences between the participants, two composite measures of the consistency of the models were built by combining the responses related to the individual model and the social model in the open-ended and closed-ended questions. The first measure was based on the frequency scale (0–3) of the open-ended questions, while the second was built on the agreement scale (1–4) of the closed-ended questions. In both cases, the social model responses were subtracted from the individual model responses. The result was a scale that could go from a minimum value to a maximum value of -3 to +3, where the highest score indicated a stronger endorsement (choice) for the individual model. Based on these consistency measures (and the original data) typologies were then created in which participants were divided into four groups for each scale (open-ended = OE; closed-ended = CE; Table B):

1. *social/no individual model*: social model with positive choices (> 1.5 for OE // > 2.5 for CE) and individual model with negative choices (≤ 1.5 for OE // ≤ 2.5 for CE);
2. *individual and social model*: both individual and social model with positive choices (> 1.5 for OE // > 2.5 for CE);
3. *individual/no social model*: individual model with positive choices (> 1.5 for OE // > 2.5 for CE) and social model with negative choices (≤ 1.5 for OE // ≤ 2.5 for CE);
4. *neither*: both individual and social model with negative choices (≤ 1.5 for OE // ≤ 2.5 for CE).

It should be noted that respondents answering the open-ended questions must construct explanations about the disability (preference), whereas the answers to the closed-ended questions express a measure of the agreement (1–4 with intermediate scores) for each item that expresses some different aspects of the three disability models (individual, social, and biopsychosocial).

**Table B: Typologies of agreement with the models derived from responses to open-ended (OE) and closed-ended (CE) questionnaires.**

|  | Negative choice range | | | | Positive choice range | | |
| --- | --- | --- | --- | --- | --- | --- | --- |
| OE | 0 | 0.5 | 1 | 1.5 | 2 | 2.5 | 3 |
| CE | 1 | 1.5 | 2 | 2.5 | 3 | 3.5 | 4 |
|  | no individual | | | | social | | |
|  |  | | | | individual and social | | |
|  | no social | | | | individual | | |
|  | neither the one nor the other | | | |  | | |

OE: levels of preference obtained by the scores from the Open-Ended Questionnaires, CE: levels of agreement obtained by the scores from the Closed-Ended Questionnaires.

**Procedure**

All interviews were conducted in the participant’s home by the author Fabio Meloni and lasted approximately 30 minutes for the adults and 45-60 minutes for the children. Children were interviewed separately from their parents. To verify children’s comprehension of the concept disabled vs. normal person, we administered the Semantic Discrimination Task. Then, children answered the following questionnaires: Child’s Interests and Activities; Disability Explanation (open-ended); Disability Explanation (closed-ended); and Child’s Disability Knowledge. The answers to the open-ended Disability Explanation questionnaire were audiotaped.

After checking the child’s questionnaires and giving their informed consent, only one of the interviewed parents completed the following questionnaires: Parent’s Education about Diversity; Disability Explanation (open-ended); Disability Explanation (closed-ended); Parent’s Interests and Activities Encouraged.

**Parent’s Protocol**

**Parent’s education about diversity**

*Materials*: Parent’s Education About Diversity Questionnaire

*Instructions*: “What follows are some explanations about the meaning of disability that adults might give to children of different ages. We would not expect adults to give exactly the same explanations. We want to know if you would give similar explanations to your own child, [*name of child involved in the experiment*]. We will show in sequence some figures that describe different situations. Imagine that your own child has witnessed situations similar to those described by the figures. In these circumstances, your child has heard about a disability, and, not knowing the meaning of the term, asks: ‘What is disability?’ For each of the possible explanations, please indicate on a 5-point scale anchored at 1, I would *never*-; 3, I *might* -; or 5, I’m very likely to give a similar explanation.”

*Statements* (n = 12): (i) Disability is a situation in which a person is different from others because God has willed it so. (ii) All people are different. When we do not accept their differences, we say that they are disabled. (iii) Being disabled or not disabled is difficult to determine, because the disability does not only depend on the functioning of the person, but also by the situation in which he or she lives. (iv) Worldwide, there are beautiful people and ugly people. Disability is a situation in which a person is not as beautiful as the others. (v) Disabled people are those who encounter many more obstacles around them. (vi) A disability is when a person is limited in doing something because of a health condition and finds obstacles in everyday life; it is also a consequence of our errors. (vii) If we behaved better, there would not be people with disabilities. (viii) In the world, those who are different from the majority often suffer injustice. Disabled people are among them. (ix) We are all a bit different; differences much depend on where we are if we function as well or not. (x) All people fall into two categories: those who command and those who do not command. The disabled are among those that do not command. (xi) A disability is the result of a disease that has forever changed our body. (xii) We all are more or less disabled; No one is completely disabled or completely able-bodied. There are, rather, different ways of functioning.

*Scale*: Unlikely/Likely from 1 to 5.

**DISABILITY EXPLANATION―OPEN-ENDED QUESTIONNAIRE**

*Materials*: Disability Explanation – Open-ended Questionnaire

*Instructions*: “Imagine that your child has asked you the following question: ‘Why does (name of the child in question, see below) have difficulties in life?’ There are no right or wrong answers to these questions only different kinds of ideas. Please write a brief answer.”

*Questions* (n = 4): (i) Giovanni is in a wheelchair because he cannot walk. In your opinion, why does he have difficulties in life? (ii) Maria is blind; she cannot see. In your opinion, why does she have difficulties in life? (iii) Paolo is autistic and he does not understand what others say. In your opinion, why does he have difficulties in life? (iv) Elena is a normal person. In your opinion, why does she have difficulties in life?

**Disability explanation―closed-ended questionnaire**

*Materials*: Disability Explanation – Closed-ended Questionnaire

*Instructions*: “We have collected some possible explanations for why some people have difficulties in life. Express the degree of your agreement or disagreement with each of the following statements knowing that: 1 means ‘strongly disagree’ and 4 means ‘strongly agree.’ There are no right or wrong answers; the best response is the most spontaneous.”

*Statements* (n = 11 x 2 masculine and x 2 feminine): (*Masculine*) (i) he’s bad; (ii) he’s ugly; (iii) God wanted it; (iv) he’s sick; (v) others mistreat him; (vi) he encounters lots of obstacles; (vii) nobody will give him a job; (viii) his body does not function well and the world makes him things difficult; (ix) the world makes him things difficult and his body does not function well; (x) he does not walk well and he does not take the bus; (xi) nobody will give him a job and he does not walk well.

*Scale*: Agreement/Disagreement from 1 to 4 with intermediate points.

PARENT’S INTERESTS AND ACTIVITIES ENCOURAGED

*Materials*: Parent’s Interests and Activities Encouraged Questionnaire

*Instructions*: “Here are some activities or interests that parents often encourage in their children. Please indicate below how much importance you place on your child engaging in each of these activities. There are no right or wrong answers to these questions, just different opinions.”

*Statements* (n = 25): (i) learn to sing or play an instrument; (ii) go to a concert; (iii) listen to music; (iv) know Jesus or other religious figures; (v) go to church or other places of worship; (vi) go to catechism or other places of religious education; (vii) pray or meditate; (viii) read the Bible or other holy books or stories of saints; (ix) usually practice sport, dance, or other physical activities; (x) go to a sporting event; (xi) play a competitive sport or participate in a dance exhibition; (xii) watch a sport on television or listen to radio programs; (xiii) attend physical education at school; (xiv) go to a museum or attend conferences; (xv) read the newspaper; (xvi) read books; (xvii) watch cultural and scientific television programs; (xviii) participate in voluntary groups; (xix) help classmates with their homework; (xx) join classmates to study or for extra-curricular activities; (xxi) give money to charities; (xxii) give money to a telethon or other social aids; (xxiii) give seat up for an elderly person on a bus; (xxiv) gather used clothes; (xv) have a good conduct mark.

*Scale*: Important/Not Important from 1 to 5.

**Child’s Protocol**

**Semantic discrimination**

*Materials*: 4 pictures (2 disabled and 2 able-bodied people); 2 cards: on one is written “normal,” on the other “handicapped.”

*Instructions*: “Here are two bags. On one is written ‘normal’ and on the other ‘disabled.’ I am going to show you some pictures and words. I would like you to put each of them into the right bag.”

*Note for the interviewer*: If the child fails in the discrimination task, repeat the task again showing just the pictures and words inserted into the erroneous bag.

**Child’s interests and activities**

*Materials*: Child’s Interests and Activities

*Instructions*: “I am going to show you some pictures several times. Each time I will show you 5 pictures. These pictures represent activities related to children. I would like for you to order them from the activity you like the most to the one that you like the least. Then I will ask you how often you do each activity. For the answer, point to here on the card if you do the activity [*the interviewer indicates the right point on the card*]: 1, Never; 3, Once or more a year; 5, Once or more a week. [*The interviewer explains that it is possible to designate intermediate points*].

*Note for the interviewer*: Warning! Administer the images in the randomized order.

*Items* (n = 20): (i) read stories form the Bible; (ii) attend worship in the Church; (iii) go to the catechism; (iv) say prayers; (v) attend parish youth club; (vi) read a book about the human body; (vii) watch a TV program about health; (viii) search the internet for news about disease; (ix) play the happy surgeon; (x) play doctor; (xi) help a classmate with homework; (xii) give seat up for an elderly person on a bus; (xiii) help push a wheelchair; (xiv) watch the TV news; (xv) read a newspaper; (xvi) visit a museum; (xvii) read fairy tales; (xviii) go to the cinema; (xix) play a sport; (xx) surf on the internet.

*Scales* (n = 2): Satisfaction 1-5; Frequency 1-5.

**Disability explanation―open-ended questionnaire**

*Materials*: Disability Explanation – Open-ended Questionnaire

*Instructions*: “I am going to ask you some questions. There are no right or wrong answers to these questions, just different kinds of ideas. I will present some people to you. These people told me they encounter difficulties in their lives. Now, think about the Target Item [*Target Item description*]. What do you think makes his or her life difficult?”

*Questions* (n = 4): (i) Giovanni is in a wheelchair because he cannot walk. In your opinion, why does he have difficulties in life? (ii) Maria is blind; she cannot see. In your opinion, why does she have difficulties in life? (iii) Paolo is autistic and does not understand what others say. In your opinion, why does he have difficulties in life? (iv) Elena is a normal person. In your opinion, why does she have difficulties in life?

*Note for the interviewer*: Audiotape the answers of the child.

**Disability explanation―closed-ended questionnaire**

*Materials*: Disability Explanation – Closed-ended Questionnaire

*Instructions*: “Inside this bag are four pictures of the four persons who we talked about previously (Giovanni, Maria, Paolo and Elena). Pull each out one at a time. In front of you there is a card with four smiles which you need to point out how much you agree with each statement that I read to you for each picture. For example, if I say: The ice cream is sweet. How much do you agree? Strongly Agree, Agree, Disagree, or Strongly Disagree. [*Make sure the child really chooses the face that corresponds to his or her level of agreement. Then, follow with a second example*]. Let me give another example. If I say: ‘The sun rises at night.’ How much do you agree? [*Even if the child shows oppositional behavior, just make sure that the choice corresponds to his or her will*]. For each picture that you pull out I will now make some statements. For each of these statements, point to the smiley which expresses your agreement or disagreement.”

*Statements* (n = 11 x 2 masculine and x 2 feminine): (*Masculine*) (i) he’s bad; (ii) he’s ugly; (iii) God wanted it; (iv) he’s sick; (v) others mistreat him; (vi) he encounters lots of obstacles; (vii) nobody will give him a job; (viii) his body does not function well and the world makes him think it is difficult; (ix) the world makes him things difficult and his body does not function well; (x) he does not walk well and he does not take the bus; (xi) nobody will give him a job and he does not walk well.

*Scale*: Agreement/Disagreement from 1 to 4 with intermediate points.

**Child’s disability knowledge**

*Materials*: Child’s Disability Knowledge

*Instructions*: “Now I am going to read some statements. In front of you there is a card with four smiles on it. You need to point out to me how much you agree with each statement that I will read to you for each picture. For each of these statements point to the smiley face that expresses your agreement or disagreement.

*Note for the interviewer*: Warning! Administer the images in the randomized order and randomized sex

*Items* (n = 8, stereotypes): (i) if a child is born disabled it is because his parents are ill; (ii) if a person becomes disabled it is because she was bad; (iii) all disabled persons are extraordinary people; (iv) disabled people cannot marry; (v) women with disabilities cannot have children; (vi) all the deaf people are also dumb (cannot speak); (vii) people with disabilities do not learn anything in school; (viii) public transportation does not allow the disabled to ride.

*Items* (n = 8, knowledge): (i) paralysis is most often caused by spinal cord injury; (ii) autistic people have difficulty in communicating with others; (iii) diabetes is a disease that can also affect children; (iv) some students with learning disabilities in writing, reading, and computing can be as smart as others; (v) all people get sick sooner or later; (vi) only disabled athletes attend the Paralympic games; (vii) when crossing the street, a blind pedestrian can make use of an acoustic traffic signal; (viii) Down Syndrome is caused by a genetic abnormality.

1. During the review process, a reviewer suggested that we should justify the use of this term as it carries the negative implication that a person with disability is not ‘normal’. Our justification is that the aim of the semantic discrimination was to verify the children’s understanding of the term ‘disability’, which was used several times during the experiment. The terms ‘non-disabled’ or ‘able-bodied’ are very uncommon in the Italian vernacular and, if we used them, we would have had to explain them, making the procedure complex and circular. In addition, using the term ‘non-disabled’ would not have allowed us to eliminate the possibility that the child did not understand the term ‘disabled’. In Italian there are no commonly used alternatives to the terms ‘normal’ and ‘non-disabled’ and in the vernacular the terms ‘handicapped’ and ‘normal’ do not imply a negative view of disability. Nevertheless, we cannot exclude the possibility that our choice of terminology did have some influence on children’s responses; however, we are confident that it did not suggest that ‘non-normal’ (i.e. disabled) equals ‘sick’. We see no reason to suppose that children would be more likely to understand ‘non-normal’ as disabled or sick, rather than with super-normal or exceptional ability (see the results for the *Disability Explanation—Open-Ended Questionnaire*). [↑](#footnote-ref-2)
